# Relationship between childcare workers’ physical literacy and support for promoting children’s physical activity

**DOI:** 10.3389/fpubh.2026.1807141

**Published:** 2026-04-13

**Authors:** Sho Honda, Misaki Matsunaga, Masahiro Matsui, Kenta Toyama, Koya Suzuki

**Affiliations:** 1 Graduate School of Health and Sports Science, Juntendo University, Chiba, Japan; 2 Juntendo Administration for Sports, Health and Medical Sciences, Tokyo, Japan; 3 Department of Child Psychiatry, Yokohama City University Hospital, Yokohama, Kanagawa, Japan; 4 Faculty of Health and Sports Science, Juntendo University, Chiba, Japan; 5 Institute of Health and Sports Science & Medicine, Juntendo University, Chiba, Japan

**Keywords:** childcare workers, early childhood education, physical literacy, Physical Literacy for Life self-assessment tool (PL4L), structural equation modeling, support for promoting children’s physical activity

## Abstract

**Introduction:**

Physical literacy (PL) is defined as “the motivation, confidence, physical competence, knowledge, and understanding required to value and take responsibility for engagement in physical activities for life.” Previous research suggests that higher parental PL is associated with positive attitudes and involvement in children’s physical activity. As approximately 90% of children in Japan attend early childhood education and care settings, the role of childcare workers in supporting children’s physical activity has become increasingly important. Therefore, we aimed to examine the characteristics of childcare workers’ PL and its association with their support for promoting children’s physical activity.

**Methods:**

Secondary data from 201 childcare workers provided by a company managing early childhood education and care facilities were used. PL was assessed using the Physical Literacy for Life self-assessment tool (PL4L). Support for promoting children’s physical activity was assessed using five items from a previous study and an additional item developed for this study. The construct validity of the support items was examined using confirmatory factor analysis, and internal consistency was assessed using Cronbach’s alpha. The Mann–Whitney U and Kruskal–Wallis tests were used to compare childcare workers’ PL scores by gender, sports club participation at each school age, and stage of change for participation in physical activity. Structural equation modeling was used to examine whether childcare workers’ PL was related to support for promoting children’s physical activity.

**Results:**

Childcare workers who had participated in sports clubs during junior high school, high school, or university tended to report higher PL scores than those who had not. PL scores differed by stage of change for participation in physical activity, with childcare workers in the Maintenance stage showing significantly higher total PL scores than those in the Pre-contemplation stage. Structural equation modeling showed an acceptable model fit and reasonable factor loadings, and the path coefficients from childcare workers’ PL to support were statistically significant.

**Discussion:**

Childcare workers’ PL appears to be associated with their experience of sports club participation and stage of change for participation in physical activity. Enhancing childcare workers’ PL may aid their support for promoting children’s physical activity.

## 1 Introduction

Physical activity in children and adolescents improves cardiorespiratory and metabolic functions, muscular strength, bone health, and mental health (1). Similar health benefits have been observed in children aged 3–6 years (2–4). Nonetheless, only 27–33% of children and adolescents meet the recommended physical activity guidelines (5). Therefore, addressing this deficit and promoting active lifestyles from early childhood are important for supporting children’s lifelong health and development.

Parental support for physical activity (e.g., enrolling children in sports clubs) is associated with children’s autonomy, physical activity, and health-related quality of life (6, 7). Therefore, parental support for children’s physical activity may be important for helping children develop regular physical activity behaviors.

According to the Organization for Economic Cooperation and Development, investment in early childhood education and care provides economic and social benefits, including reducing social inequalities, highlighting its relevance to public policy (8). In Japan, more than 90% of preschool-aged children attend kindergartens, nurseries, or other preschool settings, and basic fees for early childhood education and care for children aged 3 to 5 years were eliminated in 2020 (9, 10). Therefore, the role of childcare workers is becoming increasingly important. Previous studies have shown that childcare workers’ healthy behaviors and verbal prompts are beneficial to children (11, 12). These reports highlight the important role of childcare workers’ support in promoting children’s active lifestyles. However, the determinants of this support are poorly understood.

Childcare workers organize children’s daily activities in childcare settings, and their own motivation, engagement, and other attributes related to physical activity may relate to how they support children’s opportunities for movement. In this study, we focused on Physical literacy (PL) as one such factor, as it captures individual’s valuation of and proactive engagement in physical activity. Accordingly, childcare workers with higher PL may be more likely to provide support for promoting children’s physical activity. PL is defined as “the motivation, confidence, physical competence, knowledge, and understanding required to value and take responsibility for engagement in physical activities for life” (13). PL comprises four interrelated domains: the physical domain, which includes physical fitness and motor skills necessary to engage in physical activity; the emotional domain, which includes motivation and emotion regulation related to physical activity; the social domain, which includes cooperative and ethical behaviors demonstrated through participation in physical activity; and the cognitive domain, which includes knowledge of physical activity and ability to set appropriate goals (14, 15). While the definition and structure of PL continue to be debated internationally, this study adopts a multidimensional conceptualization that extends beyond physical competence to include motivation, cognition, social dimensions, and a sense of value and responsibility toward physical activity.

Previous research has described conceptual links between PL, physical activity, and health. In addition, associations have been demonstrated between PL and body composition, physical fitness, blood pressure, and health-related quality of life (16–18). Recent research has shown that parents with higher PL tend to hold more positive values toward physical activity and be more actively involved in supporting their children’s physical activity (19). This suggests that PL relates both to individuals’ physical activity and health and to support for promoting children’s physical activity. Higher levels of physical competence and confidence may contribute to childcare workers’ willingness to promote active play. Additionally, knowledge and understanding of physical activity can inform teaching practices and help intentionally encourage physical activity opportunities. However, while PL has been examined in parents, it has not yet been assessed in childcare workers. Considering their key role in children’s physical activity, it is important to examine whether PL is associated with their support for promoting children’s physical activity.

The aim of this study was to examine the characteristics of childcare workers’ PL and its association with their behaviors supporting children’s physical activity in childcare settings. We hypothesized that childcare workers with higher PL would provide greater support for promoting children’s physical activity in childcare settings.

## 2 Materials and methods

### 2.1 Participants

This quantitative cross-sectional study used secondary data collected by a childcare management company for its educational and operational monitoring. An online questionnaire was administered to employees between December 2024 and March 2025. In total, 234 responses were obtained. After excluding 33 participants, including those with incomplete data and those who were not childcare workers, 201 childcare workers remained for final analysis. This study adhered to the Declaration of Helsinki and was approved by the Juntendo University School of Health and Sports Science and Graduate School of Health and Sports Science Research Ethics Committee (No. 2024-174).

### 2.2 Participant characteristics

The following characteristics of childcare workers were collected: age, gender, years of employment, sports experience (elementary school, middle school, upper secondary school, and university [including junior colleges, vocational colleges, and graduate schools]), highest educational level, and stage of change model for participation in physical activity. Participants reported their age and years of employment as numeric values. For gender, participants selected either men, women, or preferred not to answer. Sports experience was assessed by asking about participation in athletics or sports clubs at each educational level they had attended. Participants’ highest educational level was recorded to determine whether they had progressed beyond high school. The stage of change model for participation in physical activity was used to assess participants’ habitual physical activity. This model describes behavior change as progressing through stages from pre-contemplation to maintenance (20). Regular physical activity was defined as exercising at least two to three times per week for 20–30 min per session. Participants then selected one of five categories representing their current stage: “I am not currently exercising and do not plan to start in the foreseeable future” (pre-contemplation); “I currently do not exercise, but I intend to exercise within the next 6 months” (contemplation); “I currently get some exercise, but not regularly” (preparation); “I currently exercise regularly, but I have only begun doing so within the past 6 months” (action); and “I currently exercise regularly and have been doing so for longer than 6 months” (maintenance).

### 2.3 Physical literacy

The Physical Literacy for Life self-assessment tool (PL4L) was used to assess childcare workers’ PL (15, 21). The PL4L consists of 16 items across four domains: six items in the physical domain (e.g., strength and stamina), four items in the emotional domain (e.g., motivation and confidence), three items in the cognitive domain (e.g., knowledge, rules, and tactics), and three items in the social domain (e.g., ethics, society, and culture). For each item, childcare workers selected one of three levels (Level 1–3) that best described their behavior. Each item was scored as 0, 1, or 2, corresponding to Levels 1, 2, and 3, respectively, and a total score was calculated for each domain. The domain scores ranged from 0 to 12 for the physical domain, 0 to 8 for the emotional domain, and 0 to 6 for both the cognitive and social domains. Each domain score was converted to a percentage using the following formula: (obtained score / maximum score) × 100. The total PL score was calculated as the mean of the four domain percentage scores.

### 2.4 Support for promoting children’s physical activity

Support for promoting children’s physical activity was assessed using six items, five of which were based on a previous study (22) and one newly developed item. Previous studies have shown that preschool children have more opportunities to engage in physical activity in environments where mobile toys (e.g., balls, tricycles) are easily accessible (23). Based on this literature, we developed an additional item assessing the provision of such equipment as a support behavior for childcare workers. These items were as follows: “How much do you usually recommend physical activity and sports to your preschool children?”; “How often do you usually take your preschool children to places where they can do physical activities and sports (school garden, park, etc.)?”; “How frequently do you observe preschool children engaging in physical activity or sports?”; “How much do you usually tell your preschool children that physical activity is good for their health?”; “How much do you usually participate in physical activities and sports with your preschool children?”; and “How often do you usually take out tools (balls, cones, etc.) when your preschool children are doing physical activities?”. Childcare workers rated the frequency of their support for promoting children’s physical activity on a five-point Likert scale: 0 (None at all), 1 (1–3 times a month), 2 (1–2 times a week), 3 (3–4 times a week), and 4 (Every day), and total scores were calculated.

Given the inclusion of a newly developed item and the adaptation of the scale to a professional childcare context, we first performed an exploratory factor analysis to assess the factor structure of the six support items. The exploratory factor analysis (maximum likelihood extraction) supported a single-factor solution (eigenvalue > 1). However, the item asking, “How much do you usually tell your preschool children that physical activity is good for their health?” Showed a low loading (0.379) and appeared conceptually distinct from the other items. A confirmatory factor analysis showed a similarly low loading for this item, leading to the removal of the item. After this item was excluded, the five items demonstrated good fit (Comparative Fit Index [CFI] = 0.981, Tucker–Lewis Index [TLI] = 0.962, Root Mean Square Error of Approximation [RMSEA] = 0.062, and Standardized Root Mean Square Residual [SRMR] = 0.028), with acceptable factor loadings (λs = 0.60–0.82) and internal consistency (*α* = 0.831) (24). Accordingly, these five items were used for analysis in this study.

### 2.5 Data analysis

First, the Kolmogorov–Smirnov test was used to assess normality. Because normality was not confirmed, non-parametric tests and the corresponding estimation methods were adopted for the analysis.

The Mann–Whitney U test was used to examine differences in total PL scores and the four domain percentage scores between gender groups and sports experience. The Kruskal–Wallis H test was used to examine differences across age, years of employment, and stage of change for participation in physical activity. Post-hoc comparisons were performed using the Bonferroni correction.

Structural equation modeling was conducted to examine the relationship between PL and support for promoting children’s physical activity. The four domain percentage scores (physical, emotional, cognitive, and social) and responses to each support item were included as observed variables. PL and support for promoting children’s physical activity were treated as latent variables. Parameter estimation was performed using the robust maximum likelihood method, which is appropriate when variables can be treated as continuous variables (25). Model fit was evaluated using the CFI, TLI, RMSEA, and SRMR. Model fit was assessed holistically using the following commonly used criteria: CFI and TLI ≥ 0.95, RMSEA and SRMR ≤ 0.08 (26, 27). All coefficients reported in the analyses were standardized.

Statistical analyses were performed using SPSS Statistics version 29 (IBM Japan, Tokyo, Japan) and Mplus version 8.11 (Muthen & Muthen, Los Angeles, USA). The level of statistical significance was set at 5%. Supplementary Table 1 provides the descriptive statistics and correlation matrix used in the structural equation modeling analysis.

## 3 Results

Participant characteristics are presented in Table 1. The mean age was 31.2 ± 11.5 years. Of 201 participants, 182 (91%) were women, 110 (55%) were in their 20s, and 99 (49%) had fewer than five years of employment. Regarding sports experience, 141 (70%) had participated in athletics or sports clubs in junior high school, and 108 (54%) in high school. Additionally, 24 (12%) were in the maintenance stage, indicating that they had engaged in regular physical activity for at least six months.

**Table 1:** Participant characteristics.

Item	*n* [%]
Age	
20s	110 [55]
30s	49 [24]
Over 40	42 [21]
Gender	
Men	18 [9]
Women	182 [91]
Prefer not to answer	1 [1]
Years of employment	
Less than 5 years	99 [49]
5 to 10 years	43 [21]
Over 10 years	59 [29]
Sports experience	
Elementary school	
Yes	123 [61]
No	78 [39]
Junior high school	
Yes	141 [70]
No	60 [30]
High school	
Yes	108 [54]
No	93 [46]
University, Junior college, professional training college, or graduate school	
Yes	46 [24]
No	149 [76]
The stages of change model for participation in PA	
Pre-contemplation	66 [33]
Contemplation	59 [29]
Preparation	44 [22]
Action	8 [4]
Maintenance	24 [12]

The final academic background of 6 participants was high school.

Differences in total PL scores and the four domain percentage scores across participant characteristics of childcare workers are presented in Table 2. The physical (H = 12.3, *p* = 0.002), emotional (H = 11.1, *p* = 0.004), and social (H = 9.9, *p* = 0.007) domains differed across age groups. Post-hoc analysis showed a higher physical domain score in participants in their 20s than in those aged over 40 years (*p* = 0.001). In the emotional domain, participants aged over 40 years scored higher than those in their 20s (*p* < 0.02) and 30s (*p* = 0.004), whereas in the social domain, they scored higher than those in their 30s (*p* = 0.007). Gender differences were not observed for total PL or domain scores (*p* > 0.05). Childcare workers employed for less than 5 years scored higher than those employed for more than 10 years in the physical domain (H = 7.9, *p* = 0.003).

**Table 2:** PL percentage scores compared by participant characteristics.

	*n*	Total		Physical domain		Emotional domain		Cognitive domain		Social domain	
		Median (IQR)	*p*	Median (IQR)	*p*	Median (IQR)	*p*	Median (IQR)	*p*	Median (IQR)	*p*
All	201	56 (50,66)	—	58 (50,67)	—	63 (50,75)	—	50 (50,67)	—	50 (50,67)	—
Age											
1: 20s	110	55 (50, 68)	0.197	58 (50, 75)	0.002	63 (50, 75)	0.004	50 (50, 67)	0.079	50 (50, 67)	0.007
2: 30s	49	54 (47, 63)		58 (50, 67)		63 (50, 63)		50 (33, 67)		50 (50, 50)	
3: Over 40	42	60 (53, 66)		50 (50, 58) a		63 (63, 75) ab		50 (50, 67)		50 (50, 67) b	
Gender											
Men	18	56 (44, 67)	0.555	58 (50, 83)	0.211	63 (50, 66)	0.559	67 (46, 63)	0.237	50 (50, 67)	0.752
Women	182	56 (50, 66)		58 (50, 67)		63 (50, 75)		50 (50, 67)		50 (50, 67)	
Years of employment											
1: Less than 5 years	99	56 (50, 68)	0.496	58 (50, 75)	0.003	63 (50, 75)	0.48	50 (50, 67)	0.229	50 (50, 67)	0.648
2: 5 to 10 years	43	55 (49, 64)		50 (50, 67)		50 (50, 63)		50 (50, 50)		50 (50, 67)	
3: Over 10 years	59	57 (50, 64)		50 (50, 58) a		63 (50, 75)		50 (50, 67)		50 (50, 67)	
Sports experience											
Elementary school											
Yes	123	57 (50, 67)	0.547	58 (50, 75)	0.001	63 (50, 75)	0.893	50 (50, 67)	0.851	50 (50,67)	0.182
No	78	55 (50, 66)		50 (50, 58)		63 (50, 75)		50 (50, 67)		50 (50,54)	
Junior high school											
Yes	141	59 (51, 68)	<0.001	58 (50, 75)	<0.001	63 (50, 75)	0.002	50 (50, 67)	0.047	50 (50, 67)	0.001
No	60	53 (47, 59)		50 (50, 58)		63 (50, 63)		50 (38, 63)		50 (50, 50)	
High school											
Yes	108	60 (52, 70)	<0.001	58 (50, 75)	<0.001	63 (53, 75)	<0.001	50 (50, 67)	0.011	50 (50, 67)	<0.001
No	93	53 (47, 60)		50 (50, 58)		63 (50, 63)		50 (33, 67)		50 (50, 50)	
University Junior college professional training college graduate school											
Yes	46	64 (22, 70)	0.002	67 (56, 75)	<0.001	63 (63, 75)	0.007	50 (50, 67)	0.073	50 (50, 67)	0.076
No	149	54 (16, 64)		50 (50, 67)		63 (50, 75)		50 (50, 67)		50 (50, 67)	
The stages of change model for participation in PA											
1: Pre-contemplation	66	53 (47, 60)	0.002	50 (50, 58)	0.004	50 (38, 63)	<0.001	50 (33, 50)	0.002	50 (50, 50)	0.002
2: Contemplation	59	56 (50, 67)		50 (50, 75)		63 (50, 75) a		50 (50, 67)		50 (50, 67)	
3: Preparation	44	60 (52, 71)		58 (50, 67)		63 (53, 75) a		50 (50, 67) a		50 (50, 67)	
4: Action	8	64 (57, 86)		58 (58, 85)		75 (63, 97) a		58 (38, 92)		50 (50, 79)	
5: Maintenance	24	61 (53, 74) a		67 (50, 75) a		69 (63, 88) a		67 (50, 79) a		67 (50, 67) a	

In the category of sports experience at universities, junior colleges, professional training colleges and graduate schools, six individuals whose highest educational attainment was high school were excluded, resulting in a total of 195 individuals.

a vs. “1,” *p* < 0.05; b vs. “2,” *p* < 0.05.

IRQ, interquartile range.

Differences in total PL scores and the four domain percentage scores were observed between childcare workers with and without sports experience across educational stages. Participants with elementary school experience had higher physical domain scores (*p* = 0.001). Those with junior high school experience had higher total PL scores (*p* = 0.001) and higher physical (*p* = 0.001), emotional (*p* = 0.002), cognitive (*p* = 0.047), and social (*p* = 0.001) domain scores. Participants with high school experience had higher total PL (*p* = 0.001) and physical (*p* = 0.001), emotional (*p* = 0.001), cognitive (*p* = 0.011), and social (*p* = 0.001) domain scores. Finally, those with university, junior college, professional training college, or graduate school experience had higher total PL (*p* = 0.002) and physical (*p* = 0.001) and emotional (*p* = 0.007) domain scores.

Total PL scores and the four domain percentage scores differed across the five stages of change for physical activity participation (Total PL: H = 17.2, *p* = 0.002; Physical: H = 15.6, *p* = 0.004; Emotional: H = 29.9, *p* = 0.001; Cognitive: H = 17.1, *p* = 0.002; Social: H = 16.9, *p* = 0.002). Post-hoc analysis showed differences between the Pre-contemplation and more advanced stages. Total PL scores were higher in the Preparation (*p* = 0.018) and Maintenance (*p* = 0.031) stages. Physical domain score was higher in the Maintenance stage (*p* < 0.007). Emotional domain scores were higher in the Contemplation (*p* < 0.031), Preparation (*p* < 0.001), Action (*p* < 0.014), and Maintenance (p < 0.001) stages. Cognitive domain scores were higher in the Preparation (*p* = 0.012) and Maintenance (*p* = 0.007) stages. Social domain score was higher in the Maintenance stage (*p* = 0.007).

**Figure 1:**
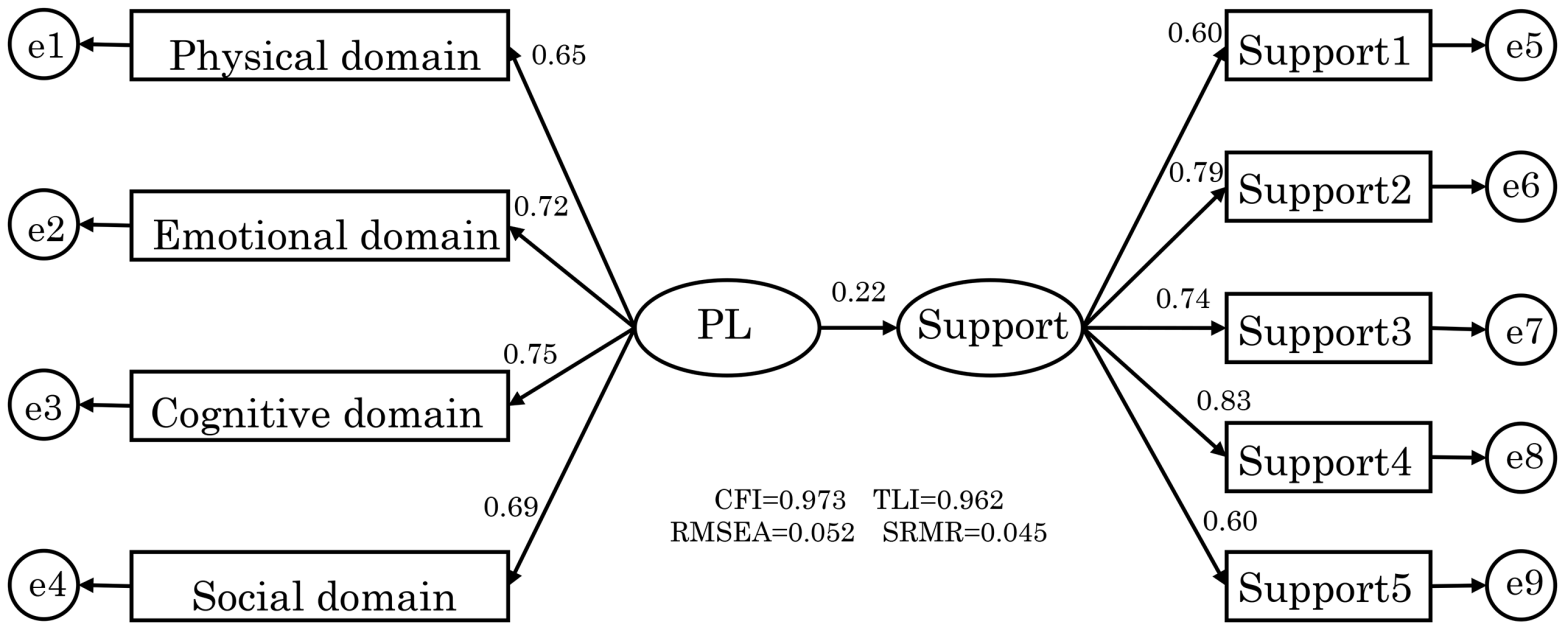
Structural equation modeling (SEM) of physical literacy and support for promoting children’s physical activity. The support for promoting children’s physical activity (Support): How much do you usually recommend physical activity and sports to your preschool children = Support1, How often do you usually take your preschool children to places where they can do physical activities and sports (school garden, park, etc.) = Support2, How frequently do you observe preschool children engaging in physical activity or sports? = Support3, How much do you usually participate in physical activities and sports with your preschool children = Support4, How often do you usually take out tools (balls, cones, etc.) when your preschool children are doing physical activities = Support5.

Results of the structural equation modeling analysis of the association between childcare workers’ PL and support for promoting children’s physical activity are shown in Figure 1. The model showed an acceptable fit to the data (CFI = 0.973, TLI = 0962, RMSEA = 0.052, and SRMR = 0.045). Factor loadings were acceptable (λs = 0.60–0.82), and PL was positively associated with childcare workers’ support for promoting children’s physical activity (βs = 0.22).

## 4 Discussion

### 4.1 PL assessment among childcare workers according to attributes

Total PL scores did not differ by childcare workers’ age, gender, or years of employment (Table 2), consistent with previous findings among teachers and university students (28, 29). In contrast, for the domain-specific percentage scores, younger childcare workers had higher scores in the physical domain, whereas older workers had higher scores in the emotional and social domains. Previous studies have reported age-related declines in physical ability and increases in self-esteem into older adulthood (30, 31). These findings suggest that age- and experience-related changes may influence domain-specific PL score.

Total PL scores were higher among childcare workers who had participated in athletic teams or sports clubs during their junior high school, high school, or university years (including junior colleges, professional training colleges, and graduate schools), which is consistent with previous studies (32). In contrast, sports participation during elementary school was not associated with total PL scores. A previous systematic review suggested that physical activity tracking is less stable during childhood than in adolescence and adulthood (33). These findings indicate that sports participation, especially from junior high school onwards, may be an important determinant of PL of childcare workers. Future research should explore not only whether individuals participate in sports but also include quantitative and contextual factors, such as sport type (individual or team), duration, intensity, and motivational context, to clarify their impact on total PL scores and domain-specific percentage scores.

Since PL includes various factors related to physical activity, it likely reflects key determinants that influence participation in sports and physical activity, such as physical fitness, self-efficacy for physical activity, knowledge about physical activity, and interpersonal understanding and coping skills (34–37). Prior examinations have shown that PL is associated with physical activity and self-efficacy (15, 38, 39), suggesting that individuals with higher PL are more likely to engage regularly in daily physical activity and sports. In the present study, childcare workers in the Maintenance stage showed higher total PL and all domain-specific percentage scores than those in the Pre-contemplation stage, consistent with previous findings. Overall, these results suggest that childcare workers’ PL appears to relate to their own physical activity and sports participation.

### 4.2 Relationship between PL and support for promoting children’s physical activity

In this study, childcare workers’ PL was positively associated with their support for promoting children’s physical activity (Figure 1). Previous studies have shown that health status, perceived physical competence, fitness perceptions, and exercise-related self-efficacy and motivation are associated with support for promoting children’s physical activity (40–42). Moreover, childcare workers who are proactive in physical activity and capable of creating appropriate play environments within their facilities are more likely to provide diverse play opportunities for children (43), highlighting the importance of practical and creative knowledge in supporting play. These findings suggest that childcare workers’ support for promoting children’s physical activity is related to multiple domains of PL, including physical, emotional, cognitive, and social domain aspects.

Nevertheless, the strength of the association between PL and support for promoting children’s physical activity was modest (*β* = 0.22). Although statistically significant, the effect size suggests that PL is associated with support for promoting children’s physical activity, but does not fully explain this support. These findings, therefore, provide exploratory evidence of association. Previous studies have shown that organizational and resource-related constraints within childcare settings, such as limited staffing, time, and financial resources, act as major barriers to children’s engagement in physical activity (44, 45). In addition, environmental factors, including the availability of play equipment and indoor space, have been associated with children’s physical activity (46, 47). These findings suggest that childcare workers’ PL alone may be insufficient to support children’s physical activity in the presence of organizational and environmental constraints. Accordingly, efforts to promote children’s physical activity in childcare settings may benefit from considering individual attributes, such as PL, and organizational and environmental factors. Future research could build on present findings by incorporating organizational and environmental factors, such as staffing levels, time allocated for active play, availability of play equipment, and physical space, into analytical models.

### 4.3 Social significance and contributions

This study showed an association between childcare workers’ PL and their support for promoting children’s physical activity. Previous research suggests that, within childcare settings and pre-service education, opportunities to develop knowledge, skills, and practical training related to physical activity may be limited among childcare workers (45, 48). These findings indicate that there may be room to further develop educational approaches for preparing childcare workers to support children’s physical activity within current educational systems. In this context, PL may offer a useful perspective for designing and evaluating educational programs related to physical activity for childcare workers. Educational approaches informed by the PL concept may help to promote childcare workers’ own physical activity, as well as the quality of support for children’s physical activity.

### 4.4 Limitations

This study has several limitations. First, the cross-sectional design limits the ability to determine causal relationships between childcare workers’ PL and their support for promoting children’s physical activity. While higher PL may lead to increased support behaviors, a reverse association is also possible; childcare workers who frequently engage in such support may enhance their PL attributes through practical experience. Therefore, future longitudinal and intervention studies are needed to clarify these causal and reverse causal relationships. Second, we did not fully examine the educational policies or environments of the participating childcare facilities. Future research that incorporates these factors can provide a more comprehensive understanding of the determinants of both PL and support for promoting children’s physical activity. Third, the sample was limited to childcare workers employed by a single company operating facilities in the Tokyo metropolitan area. Organizational culture, educational philosophies, curriculum policies, and sociocultural contexts may influence both PL and support practices. Therefore, the generalizability of the findings should be interpreted with caution. Fourth, because support for promoting children’s physical activity, the stage of change for participation in physical activity, and PL were measured through self-report, the findings may be affected by social desirability bias. In addition, although participation in physical activity was not included in the PL assessed in this study, assessing both factors with questionnaires may introduce common-method bias. These biases may have influenced the magnitude of the associations observed. Future research may benefit from employing multi-method designs, such as observational data in childcare settings, device-based measures of physical activity, and performance-based assessments of PL for both childcare workers and children. Nevertheless, because individual childcare workers interact with multiple children daily, responses from 201 participants may still reflect aspects of everyday childcare practices.

## 5 Conclusion

This study revealed an association between childcare workers’ PL and their support for promoting children’s physical activity in childcare settings. Attributes such as sports participation during junior high school, high school, and university (including junior colleges, professional training colleges, and graduate schools), as well as the stages of change for physical activity participation, were associated with PL. These findings suggest that PL may be a useful perspective for supporting childcare workers in promoting children’s physical activity.

## Data Availability

The data analyzed in this study is subject to the following licenses/restrictions: the raw data supporting the conclusions of this article will be made available by the authors, without undue reservation. Requests to access these datasets should be directed to Koya Suzuki, ko-suzuki@juntendo.ac.jp.
